# High sodium diet converts renal proteoglycans into pro-inflammatory mediators in rats

**DOI:** 10.1371/journal.pone.0178940

**Published:** 2017-06-08

**Authors:** Ryanne S. Hijmans, Pragyi Shrestha, Kwaku A. Sarpong, Saleh Yazdani, Rana el Masri, Wilhelmina H. A. de Jong, Gerjan Navis, Romain R. Vivès, Jacob van den Born

**Affiliations:** 1 Department of Internal Medicine, Division of Nephrology, University Medical Center Groningen, University of Groningen, Groningen, The Netherlands; 2 Institut de Biologie Structurale (IBS), Université Grenoble Alpes, Grenoble, France; 3 Department of Laboratory Medicine, University Medical Center Groningen, University of Groningen, Groningen, The Netherlands; University Medical Center Utrecht, NETHERLANDS

## Abstract

**Background:**

High dietary sodium aggravates renal disease by affecting blood pressure and by its recently shown pro-inflammatory and pro-fibrotic effects. Moreover, pro-inflammatory modification of renal heparan sulfate (HS) can induce tissue remodeling. We aim to investigate if high sodium intake in normotensive rats converts renal HS into a pro-inflammatory phenotype, able to bind more sodium and orchestrate inflammation, fibrosis and lymphangiogenesis.

**Methods:**

Wistar rats received a normal diet for 4 weeks, or 8% NaCl diet for 2 or 4 weeks. Blood pressure was monitored, and plasma, urine and tissue collected. Tissue sodium was measured by flame spectroscopy. Renal HS and tubulo-interstitial remodeling were studied by biochemical, immunohistochemical and qRT-PCR approaches.

**Results:**

High sodium rats showed a transient increase in blood pressure (week 1; p<0.01) and increased sodium excretion (p<0.05) at 2 and 4 weeks compared to controls. Tubulo-interstitial T-cells, myofibroblasts and mRNA levels of VCAM1, TGF-β1 and collagen type III significantly increased after 4 weeks (all p<0.05). There was a trend for increased macrophage infiltration and lymphangiogenesis (both p = 0.07). Despite increased dermal sodium over time (p<0.05), renal concentrations remained stable. Renal HS of high sodium rats showed increased sulfation (p = 0.05), increased L-selectin binding to HS (p<0,05), and a reduction of sulfation-sensitive anti-HS mAbs JM403 (p<0.001) and 10E4 (p<0.01). Hyaluronan expression increased under high salt conditions (p<0.01) without significant changes in the chondroitin sulfate proteoglycan versican. Statistical analyses showed that sodium-induced tissue remodeling responses partly correlated with observed HS changes.

**Conclusion:**

We show that high salt intake by healthy normotensive rats convert renal HS into high sulfated pro-inflammatory glycans involved in tissue remodeling events, but not in increased sodium storage.

## Introduction

High sodium intake is known to aggrevate renal disease [1–3]. We previously showed that high dietary sodium can cause tissue remodeling and a decline in kidney function [4–7]. Moreover, moderate sodium restriction has shown to have a protective effect in chronic kidney disease (CKD) and combining sodium restrictive dietary measures with common treatment regimens for CKD and proteinuria, enhanced the therapeutic effects [4,5,8]. The mechanism of this renoprotective effect of sodium restriction has always been ascribed to the decrease in blood pressure [9–11]. Although this “dietary sodium–blood pressure” pathway of causative renal damage is well-known and is thoroughly documented, new research also suggests involvement of a “dietary sodium–blood pressure independent” pathway as well, leading to renal damage [12]. Nonetheless, the exact mechanisms behind this blood pressure independent pathway are still unknown. Titze *et al*. showed that excess sodium can be stored in the skin, becoming osmotically inactive, thereby creating a buffering option during high sodium conditions [13]. Furthermore, they showed that osmotically inactive Na^+^ storage in the skin is an active process characterized by an increased glycosaminoglycan (GAG) content and sulfation in the reservoir tissue, leading to dermal tissue remodeling demonstrated by increased lymphangiogenesis and macrophage and T-cell influx [14,15]. Proteoglycans (PGs) are glycoconjugates consisting of a protein core, to which highly anionic GAGs are covalently attached [16]. They are abundantly expressed and can be found in extracellular matrix and on cell membranes [17]. Dictated by their sulfation pattern, GAGs interact with various proteins and orchestrate biological processes like cell-cell and cell-matrix interactions, growth factor signaling cascades, chemokine and cytokine activation, tissue morphogenesis, cell migration and proliferation, and wound healing [18]. Proteoglycans act as a scaffold/platform for growth factors, cytokines, and most prominently chemokines [16,19]. In previous studies, we have shown that critical pro-inflammatory modifications of renal heparan sulfate (HS) proteoglycans result in tissue remodelling responses (inflammation and fibrosis) after ischemia/reperfusion, renal transplantation and proteinuria [20–22]. Furthermore, scarce information suggests that the amount and type of cations bound to glycosaminoglycans modulate its 3D-structure and biological properties [23–25]. We assume that HS changes also might occur upon high dietary sodium intake. We therefore hypothesize that high dietary sodium intake convert renal HS into a pro-inflammatory phenotype, able to bind more sodium and orchestrate influx of inflammatory cells, fibrosis and lymphangiogenesis. To this end normotensive healthy male rats were fed with a high sodium diet and compared to sex- and age-matched rats on control diet, followed by evaluation of renal HS proteoglycans, sodium content and renal tissue remodelling. Here, we report an increased sulfation of renal HS upon high salt diet, resulting in the conversion of renal HS into pro-inflammatory glycans involved in tissue remodeling events, however not functioning as a storage depot for sodium.

## Materials and methods

### Experimental design of animal experiment

Fifteen three-month old male normotensive salt-insensitive Wistar rats were randomly divided into three groups. Rats included in the first group (N = 5) served as healthy controls and received normal rat chow diet for four weeks and were sacrificed afterwards. Rats in the second and third group (N = 5 each) received normal chow containing 8% NaCl (AB diets, Arie Blok B.V., Woerden, The Netherlands) and 1% NaCl in drinking water for two weeks and four weeks prior to sacrifice. Body weight and blood pressure was measured at baseline, 1 week, 2 weeks, 3 weeks and 4 weeks with the Cardiocap/5 (Datex-Ohmeda, Newark, USA). We used the non-invasive blood pressure recordings by the Cardiocap/5 device using the tail cuff method in awake rats. The rats were trained for two weeks before the experiment started and underwent the measurements during the experiment without stress and restrainers. Rats were sacrificed by cervical dislocation under general anesthesia. At the moment of sacrifice, organs were harvested after saline perfusion. Kidneys, abdominal skin and ears of all fifteen rats were taken and cryo-preserved. The kidneys were used for immunohistochemistry, binding assays, sodium measurements and qRT-PCR. The abdominal skin and the ears were used for sodium measurement. Blood plasma was collected at 2 weeks and 4 weeks, urine at baseline, 2 weeks and 4 weeks. At baseline, 2 and 4 weeks rats were placed in metabolic cages for 24 h urine collection and the measurement of food and water intake. Creatinine in plasma and urine was measured by an enzymatic UV assay (Roche Modular P).

The experiment was carried out under a protocol, which was approved by the Animal Care Committee of the University of Groningen (licence number 6318A).

### Immunohistochemistry

Staining was performed on 3-μm-thick formalin-fixed paraffin sections after deparaffinization in xylene and rehydration in alcohol series. Antigen retrieval was done for 15 min in a microwave oven in Tris/EDTA buffer pH:9.0, citrate buffer pH:6.0, or overnight at 80°C in Tris/HCl buffer pH:8.0. Endogenous peroxidase activity was blocked with 0.3% hydrogen peroxide. Sections were incubated for 1 h or overnight at 4°C with the following primary antibodies: mouse anti-human α-SMA (clone 1A4, Sigma-Aldrich, St Louis, USA), goat anti-collagen III (cat. no. 1330–01, Southern Biotech, Birmingham, USA), rabbit anti-rat CD3 (clone A0452, Dako, Glostrup, Denmark) for T cells, mouse anti-rat CD68 (clone ED1, AbD Serotec, Oxford, UK) for macrophages and mouse anti-rat podoplanin (cat. no. 11–035, Angio Bio, Del Mar, USA) for lymphatic vessels. After this step, the sections were incubated with secondary and tertiary antibodies diluted in PBS/1% BSA and 1% normal rat serum. We used rabbit anti-mouse Ig horseradish peroxidase (HRP), goat anti-rabbit Ig HRP, goat anti-mouse Ig HRP, rabbit anti-goat Ig HRP and swine anti-rabbit Ig HRP (all from Dako, Glostrup, Denmark). As negative controls, the primary antibodies were replaced by PBS/1% BSA. Bound antibodies were visualized by aminoethylcarbazole (AEC) or by 3,3′-diaminobenzidine (DAB) (Sigma-Aldrich, St Louis, USA) and then counterstained with diluted hematoxylin. Tissue sections were scanned by a NanoZoomer HT (Hamamatsu Photonics K.K., Shizuoka Pref., Japan). ED1+ macrophages, CD3+ T-cells and podoplanin+ lymphvessels were manually counted in 30 cortical interstitial fields per kidney. The expression of collagen type III was measured by using an automatic quantification method using ImageJ 1.41 (Rasband, W.S., U.S. National Institutes of Health) and expressed as a % positively stained area.

### Immunofluorescence

In order to investigate eventual changes in structure of renal HS by high salt diet, ligand binding assays for MCP-1 and L-selectin were performed. These two proteins recognize two different binding sites of HS. To detect the changes in capacity of renal proteoglycans to bind with MCP-1 and L-selectin, 4μm thick renal cryosections were fixed by paraformaldehyde, followed by incubation with MCP-1 (4ug/ml, Peprotech, Rocky Hill, USA) and L-selectin-Fc (1:100, [26]) in PBS+0.2M NaCl and TBS (final NaCl concentration is 0.18M) respectively [26]. Salt concentration was adjusted to achieve critical binding in control tissue in order to detect loss or gain of binding in kidneys from sodium-fed rats. Mouse anti human MCP-1 (1:400, Peprotech, Rocky Hill, USA) was used as the primary antibody and rabbit anti mouse IgG HRP (1:100, DAKO, Heverlee, Belgium) was used as the secondary antibody for the MCP-1 binding assay. Similarly, rabbit anti human IgM HRP (1:100, DAKO) was used as the conjugated antibody for L-selectin binding assay. For the immunohistochemistry analysis of hyaluronan and versican 4μm thick renal cryosections were fixed with acetone and endogenous peroxidase activity was blocked with 0.03% hydrogen peroxide. Endogenous biotin binding sites were blocked by an Avidin/Biotin blocking step in case of hyaluronan. Sections were incubated for 1 hour with biotinylated hyaluronan binding protein (1:20, HABP, Seikagaku, Tokyo, Japan) and Versican (1:8000, ITK Diagnostics B.V., Uithoorn, the Netherlands), respectively. Streptavidin-C3Y (1:50, Invitrogen, Carlsbad, USA) and goat anti-rabbit HRP (1:100, DAKO, Heverlee, Belgium) were used as conjugates for these stainings.

We performed a double staining for L-selectin/IgM (2004; 1:25, [26]) and JM13 (1991; 1:50, [27]). After washing with TBS, tissues were incubated with rabbit anti-human IgM HRP and goat anti-mouse IgM FITC conjugates (both 1:100, DAKO, Heverlee, Belgium). HRP activity was visualized using the TSATM Tetramethylrhodamine System (PerkinElmer LAS Inc., Waltham, USA). DAPI solution was applied to the sections and incubated for 10 minutes for nuclear staining. The same protocol was used for negative controls, however the L-selectin-Fc or MCP-1 incubation step was omitted. Stainings for hyaluronan and versican, and binding of L-selectin and MCP-1 was evaluated on a Leica DM4000B equipped for immunofluorescence, and with a DFX345FX camera using a LAS software package. Eight pictures at 200x magnification per kidney were taken followed by digital quantification by ImageJ 1.41 (Rasband, W.S., U.S. National Institutes of Health) and expressed as a % positively stained area.

For anti-HS mAbs JM403 and 10E4, we randomly selected 10 glomeruli per kidney and measured the intensity of the staining of the glomerulus (JM403) and Bowman’s capsule (10E4) automatically (correcting for background) using ImageJ 1.41 (Rasband, W.S., U.S. National Institutes of Health).

### Measurements of renal and dermal sodium

Cortical renal tissue was obtained by cutting a triangular shaped sample from the cortical area of the kidney. Abdominal skin tissue was obtained and shaved before sodium measurements. Samples were cut in half and the wet weight of each part was measured. Per sample, both halves were dried overnight at 80°C, dry weight was measured and one of the halves solved in pure nitric acid (Sigma-Aldrich, St Louis, USA) for sodium measurements. The second half of the sample was used to calculate the amount of protein per sample by measuring the nitrogen content according to Dumas using the Gerhardt Dumatherm Nitrogen/Protein analyser (C. Gerhardt UK Ltd, Northamptonshire, UK). Sodium concentrations were measured by atomic absorption (flame) spectrometry (Thermo M Series AA Spectrometer, Thermo Fisher Scientific, Waltham, USA) and expressed per dry weight and per nitrogen content.

### Extraction and purification of GAGs from renal samples

From each rat, ~30 mg frozen kidney tissue was collected. The five renal samples per group were pooled, resulting in three pooled samples (control, 2 weeks high sodium diet and 4 weeks high sodium diet). Extraction and purification of HS was performed as described previously [28–30]. Briefly, renal tissues were re-suspended in 50 mM Tris Buffered Saline (TBS), 2 mM EDTA, 6M Urea and mechanically disrupted with a Potter grinder. After recovery of the supernatant, the pellet was washed again in 50 mM TBS, 2 mM EDTA, 6M urea and centrifuged. Both supernatants were pooled and dialysed against 25 mM Tris, 5 mM EDTA pH 7.8. Proteins were then degraded by pronase digestion (2 mg/ml of pronase, final concentration, incubation for 24h at 37°C), and precipitated by addition of ice-cold trichloroacetic acid (TCA, 5% v/v final concentration) and incubated at 4°C for 1h. The samples were centrifuged, the pellets were treated again with TCA. Supernatants from both TCA treatments were collected, pooled, and supplemented with diethylether (50% v/v final concentration). After shaking, the organic upper phase was discarded and diethylether washing was repeated 4 times. The pH from the recovered aqueous phase was then adjusted to 7 by addition of 1M sodium carbonate, and residual diethyl ether was eliminated by leaving the samples overnight in a low-pressure environment.

The sample was then applied to a DEAE-Sephacel column (2 mL) equilibrated in 20 mM phosphate pH 6.5. After extensive washing with 20 mM phosphate, 0.3 M NaCl pH 6.5, GAG chains were step-eluted with 20 mM phosphate, 1 M NaCl pH 6.5. Recovered samples were desalted over a Pd-10 column, lyophilized, and stored at − 20°C prior to analysis.

### Disaccharide analysis of HS by reverse-phase ion-pair high-performance liquid chromatography (RPIP-HPLC) analysis

GAG samples were dissolved in 100 mM sodium acetate, 0.5 mM CaCl_2_, pH 7.1 and HS was exhaustively digested into disaccharides by incubation with heparinase I (10 mU, Grampian enzymes, Orkney, UK) overnight at 30°C, followed by a second incubation with heparinase II and heparinase III (10 mU each, Grampian enzymes) for 24 h at 37°C. Compositional analysis was performed by RPIP-HPLC, as described previously [31]. Samples were applied to a Luna 5μ C18 reversed phase column (4.6 × 150 mm, Phenomenex) equilibrated at 0.5 mL/min in 1.2 mM tetra-*N*-butylammonium hydrogen sulfate and 8.5% acetonitrile, and then resolved using a NaCl gradient (0–8 mM in 10 min, 8–30 mM in 1 min, 30–56 mM in 11.5 min, 56–106 mM in 1.5 min, and 106 mM for 6 min) calibrated with disaccharide standards (Iduron, Alderley Edge, UK). On-line post-column disaccharide derivatization was achieved by the addition of 2-cyanoacetamide (0.25%) in NaOH (0.5%) at a flow rate of 0.16 mL/min, followed by fluorescence detection (excitation 346 nm, emission 410 nm). Disaccharide analyses of each pool were performed in triplicate.

### Statistical analyses

Statistical analysis was performed using SPSS 23.0 (SPSS Inc., Chicago, IL, USA) and GraphPad Prism 5.0 (GraphPad Software Inc., La Jolla, CA, USA) was used to construct graphs and figures. Statistical differences between two groups were tested using Mann-Whitney-U test. Kruskall-Wallis test was used to compare multiple groups at the same time point. Correlations between independent variables were tested using Spearman Rank correlation. Partial correlation was used to correct for possible confounders. Statistical differences of p<0.05 were considered significant.

## Results

Rats who received a high sodium diet (N = 10 at two weeks and N = 5 at four weeks) did not differ in body weight compared to control rats (N = 5; Table 1), despite the fact that high sodium rats ate more. High sodium rats did not show significant differences in blood pressure over time compared to controls (AUC blood pressure), although a short hypertensive peak could be observed after the one-week diet, suggesting that the high sodium rats restored stable blood pressure and plasma sodium, by increasing their sodium excretion. High salt rats increased their water intake and urine production (for all these parameters, see Table 1). Importantly, there were no significant differences in creatinine clearance and proteinuria between high sodium rats and control rats up to four weeks, indicating that within this time frame renal function is not influenced by high dietary sodium intake (Table 1).

**Table 1:** General parameters.

	Controls N = 5, all timepoints	High sodium rats N = 10 at T0, T1, T2 N = 5 at T3, T4	P-value
**Body weight (g)**			
T0	386 [369–392]	383 [353–404]	0.759
T1	394 [380–408]	390 [361–417]	0.500
T2	426 [414–446]	413 [381–442]	0.125
T3	428 [408–443]	430 [408–445]	0.402
T4	444 [424–455]	440 [413–445]	0.209
**Food intake (g/24h)**			
T0	1.0 [0.8–7.6]	1.0 [0.8–9.8]	0.806
T2	0.0 [0.0–4.9]	3.5 [0.4–11.4]	0.041
T4	0.5 [0.4–14.1]	7.4 [5.6–14.2]	0.076
**Systolic blood pressure (AUC in arbitrairy units)**			
T0-2	17.5 [10.5–19.5]	10.5 [5.0–39.2]	0.624
T0-4	30.8 [14.2–43.8]	35.3 [24.0–61.2]	0.347
**Systolic blood pressure (mmHg)**			
T0	152 [138–158]	155 [113–167]	0.624
T1	132 [125–160]	164 [143–190]	0.007
T2	143 [140–147]	148 [124–180]	0.297
T3	150 [141–162]	155 [130–203]	0.754
T4	153 [125–168]	172 [155–192]	0.076
**Water intake (ml/24h)**			
T0	11.7 [4.7–25.6]	9.6 [4.5–27.6]	0.713
T2	11.4 [1.4–20.5]	23.6 [14.7–35.7]	0.020
T4	17.7 [2.7–26.3]	28.2 [13.6–36.8]	0.076
**Urine production (ml/24h)**			
T0	14.0 [5.0–26.0]	13.0 [9.5–19.0]	1.000
T2	16.0 [8.0–26.0]	25.5 [14.0–29.5]	0.057
T4	21.0 [11.5–22.0]	26.0 [16.0–31.0]	0.047
**Sodium excretion (mmol/24h)**			
T0	0.56 [0.31–0.95]	0.67 [0.37–2.09]	0.440
T2	0.67 [0.60–0.74]	5.34 [2.41–9.06]	0.002
T4	0.62 [0.29–2.52]	6.21 [1.90–9.67]	0.016
**Plasma sodium (mmol/L)**			
T2	140.0 [138.0–141.0]	140.5 [137.0–143.0]	0.352
T4	141.0 [137.0–142.0]	142.0 [140.0–144.0]	0.242
**Creatinine clearance (mL/min)**			
T2	4.8 [4.6–4.9]	3.9 [3.5–4.7]	0.052
T4	3.9 [1.7–4.5]	4.8 [3.8–8.3]	0.117
**Albuminuria (mg/24h)**			
T0	6.8 [1.4–65.5]	7.3 [0.9–56.0]	0.806
T2	8.7 [2.2–95.4]	12.8 [1.9–101.0]	0.739
T4	5.4 [1.7–84.9]	17.6 [2.9–137.6]	0.251

Measurement of general parameters in controls rat on normal chow diet (N = 5 at all time-points) and in rats on high salt diet (N = 10 on T0, T1, T2; N = 5 on T3, T4). Time-points represent weeks. Values are expressed in median [range] and statistical testing was done by performing Mann-Whiney-U test. P<0.05 is considered to be a significant difference between groups at a certain time-point.

### High sodium diet induces tubulo-interstitial remodeling

**Fig 1:**
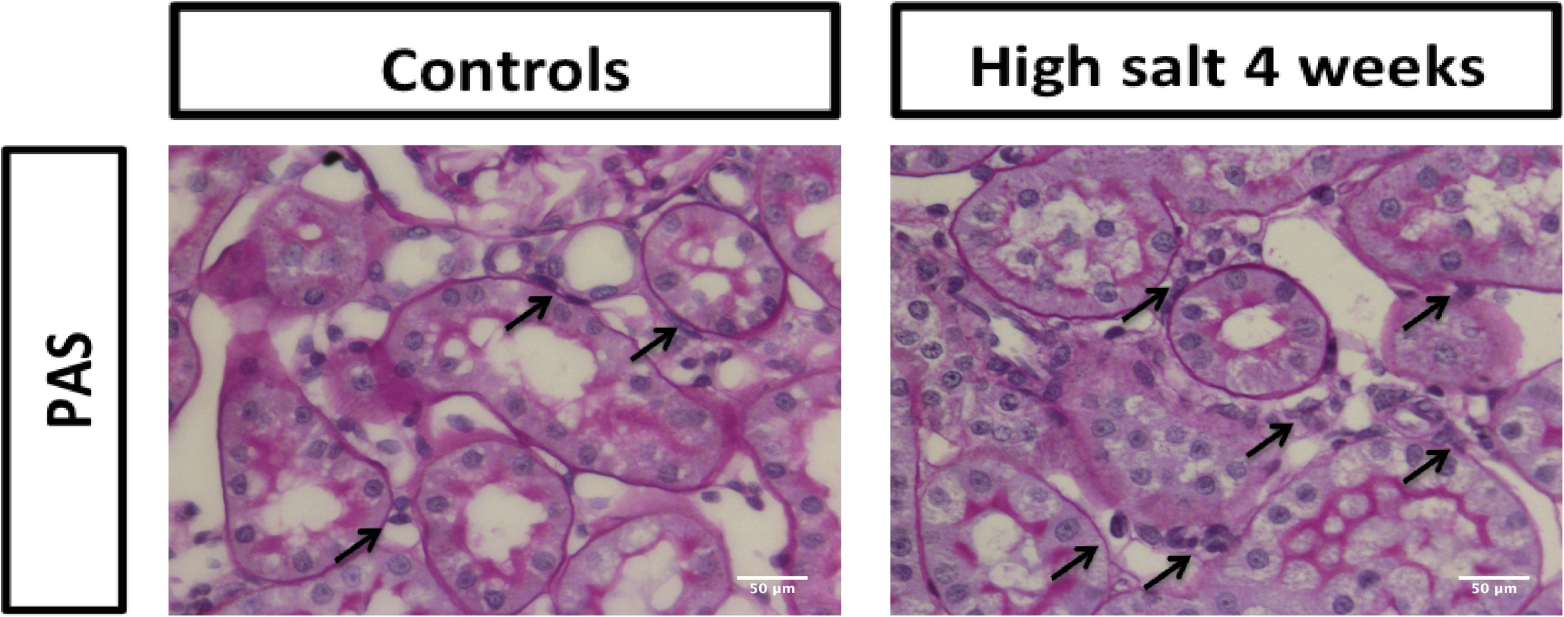
PAS staining of kidney of a control rat (left) and high salt fed rat (right). No apparent fibrosis was observed upon high salt feeding, however, we noted quite some accumulation of tubulo-interstitial cells (arrows) in the high salt kidneys. Scale bar represents 50 μm.

**Fig 2:**
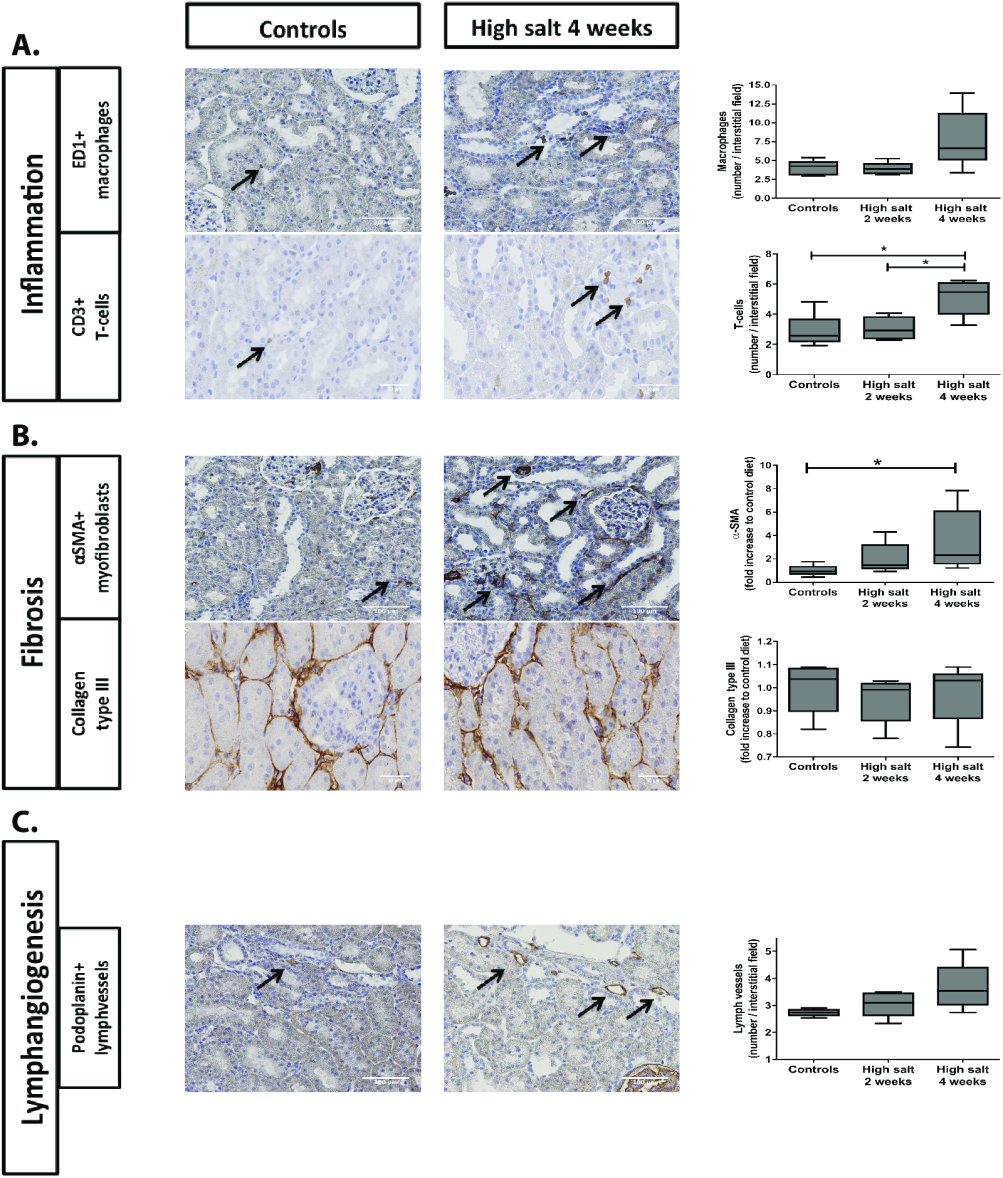
**Influence of high salt diet on renal tubulo-interstitial inflammation (A), fibrosis (B) and lymphangiogenesis (C).** IHC was done for ED1+ macrophages (A), CD3+ T-Cells (A), α-SMA+ myofibroblasts (B), collagen type III (B) and podoplanin+ lymphvessels (C). Scale bar represents 50 or 100 μm. Statistical differences between groups (N = 5/group) were tested using Kruskall-Wallis test. *p<0.05.

In order to investigate to which extent high sodium diet affects tubulo-interstitial remodeling responses, we first evaluated a regular PAS staining. No apparent tubulo-intersitial or glomerular fibrotic responses were noted, however in the high salt fed rats we observed quite some accumulation of cells in the tubulo-interstitial areas (Fig 1). To better evaluate tubulo-interstitial tissue remodeling, we specifically evaluated markers for inflammation, fibrosis and lymphangiogenesis. In terms of inflammation, the influx of ED1+ macrophages showed an increasing trend over time in high sodium rats compared to controls (Fig 2A; p<0.07). The influx of CD3+ T-cells is increased in high sodium rats at four weeks compared to controls (p<0.05) and compared to high sodium rats at two weeks (Fig 2A; p<0.05). Focusing on fibrosis, an increased accumulation of α-SMA+ myofibroblasts was found in high sodium rats at both two (NS; p = 0.12) and four weeks (Fig 2B; p<0.05). The high salt diet did not have any effect on renal collagen type III protein expression after two or four weeks (Fig 2B). Podoplanin+ lymph vessels showed an increasing trend over time in high sodium rats compared to controls, however there were no significant differences between the 3 groups (Fig 2C; p<0.07).

**Fig 3:**
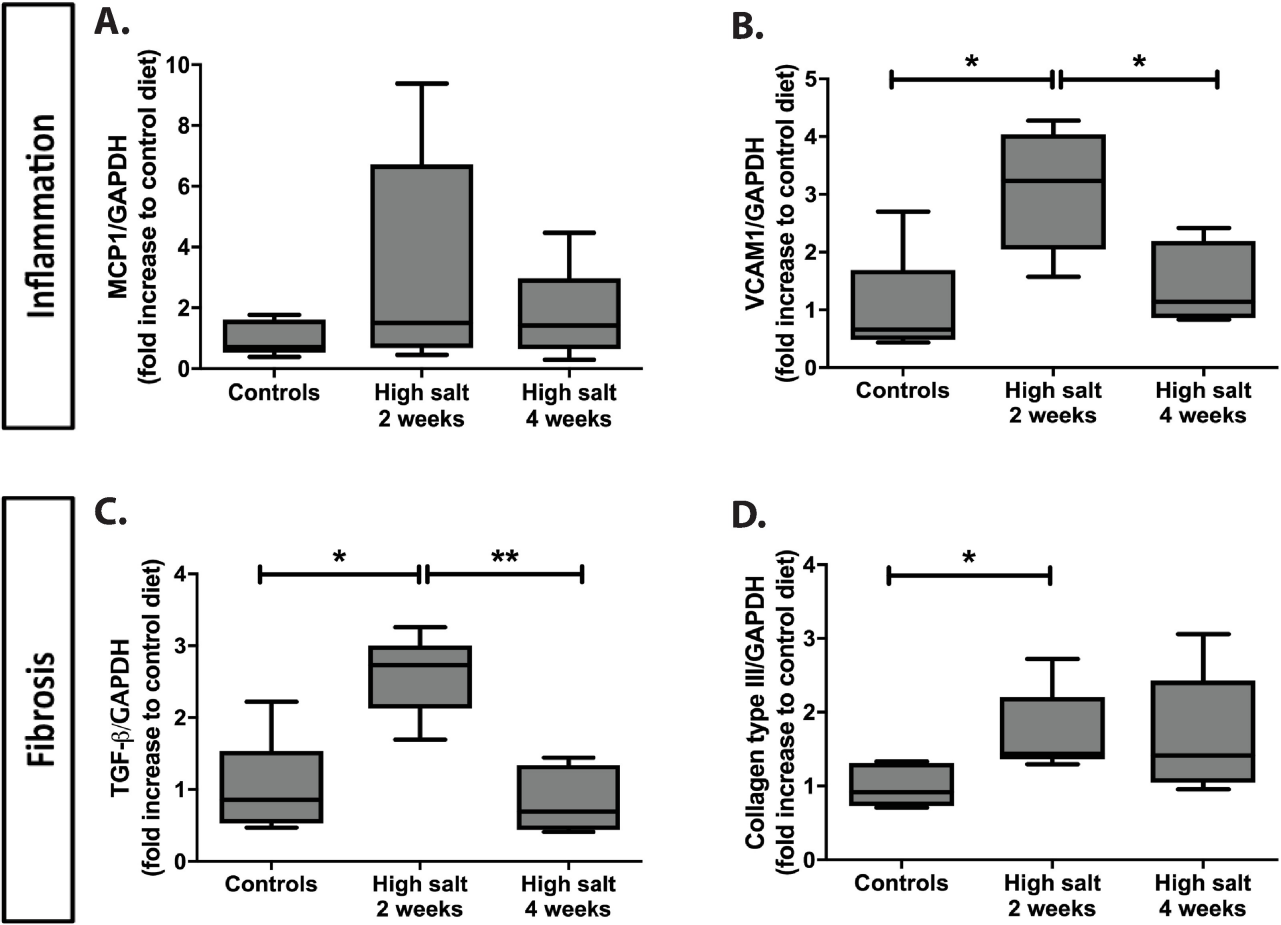
**Influence of high salt diet on the mRNA expression of inflammatory (A, B) and fibrotic markers (C, D).** mRNA expression relative to GAPDH was measured for inflammatory markers (A; MCP-1, B; VCAM1) and fibrotic markers (C; TGF-β, D; collagen type III) and expressed as fold increase to control diet. Statistical differences between groups (N = 5/group) were tested using Kruskall-Wallis test. *p<0.05.

In addition, we analyzed tubulo-interstitial remodeling phenomena in renal tissue by mRNA expression levels of inflammatory and fibrotic markers. While changes in tissue remodeling (Fig 2) were predominantly apparent after 4 weeks, we already found most changes on mRNA level after 2 weeks. MCP-1 showed no significant differences between groups (Fig 3A). VCAM1 showed a significant increase in high sodium rats at two weeks compared to controls (p<0.05) and a significant return in the high sodium rats at four weeks compared to high sodium rats at two weeks (Fig 3B; p<0.05).

Although at the protein level collagen type III expression was not different in high sodium rats compared to controls, on mRNA expression level, both TGF-β and collagen type III expression was increased compared to controls (Fig 3C and 3D; both p<0.05). TGF-β expression significantly decreased in high sodium rats at four weeks compared to high sodium rats at two weeks (p<0.01) and collagen type III mRNA expression apparently remained increased at four weeks compared to controls (NS; p<0.08).

### Dermal and renal sodium content

**Fig 4:**
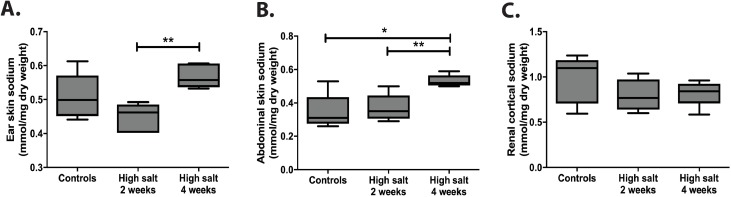
**Sodium concentrations in ear skin (A), abdominal skin (B) and cortical kidney tissue (C).** Sodium concentrations were measured by flame spectrometry and expressed in mmol sodium per mg dry weight. Statistical differences between groups (N = 5/group) were tested using the Kruskall-Wallis test. *p<0.05 and **p<0.01.

Since Titze et al. showed that excess sodium could be stored non-osmotically in the skin [13], we aimed to measure dermal and renal sodium. Dermal sodium concentrations expressed per mg dry weight in ear skin of high sodium rats did not significantly differ from controls at baseline, however, increased over time, (Fig 4A; week 4 vs week 2: p<0.01). We also measured dermal sodium concentrations in abdominal skin, which showed significantly higher sodium concentrations expressed per dry weight in high sodium rats compared to controls at four weeks (p<0.05) and over time in high sodium rats (Fig 4B; p<0.01). When we compared these findings with cortical renal sodium content (Fig 4C), no significant differences between high sodium rats and controls or significant differences over time were found. The same findings were obtained when sodium concentrations were expressed per mg protein as calculated form nitrogen content of the samples (data not shown).

Therefore, we found no evidence that excess sodium is being stored in the kidney in contrast to dermal tissue.

### Structural and functional HS changes by high salt diet

**Fig 5:**
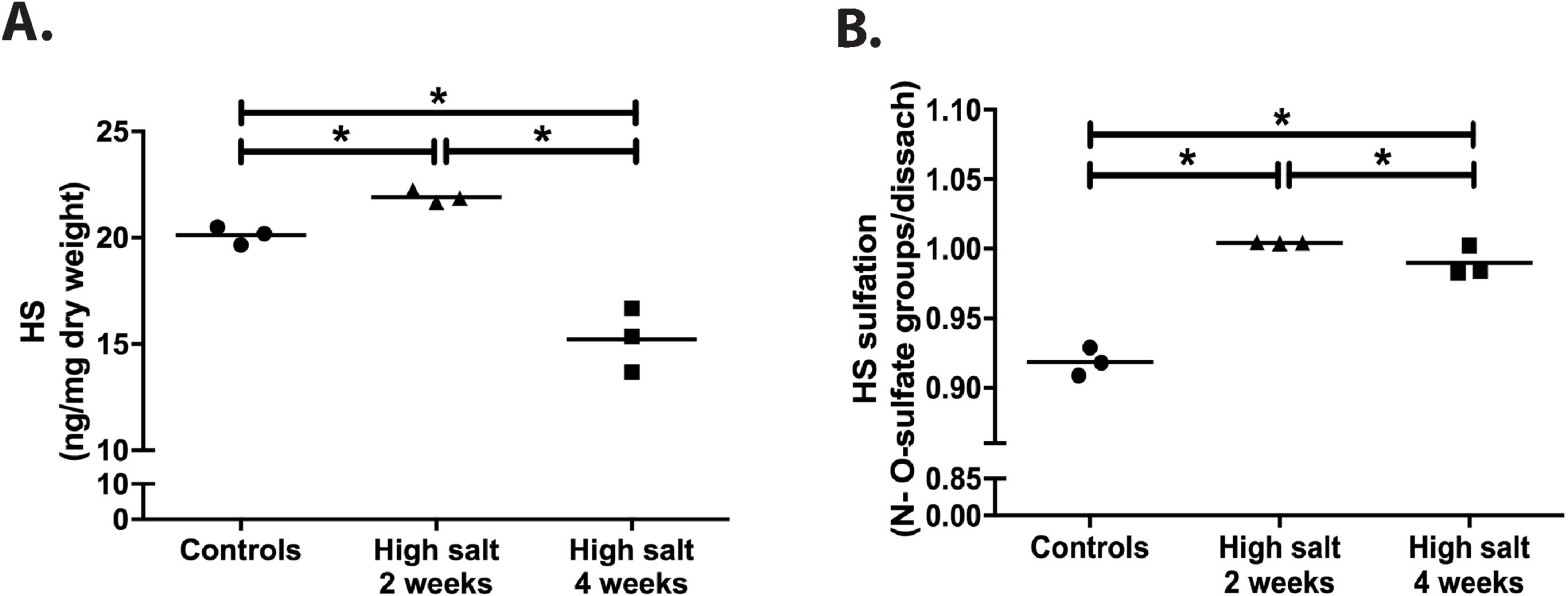
High salt diet induced changes in amount and sulfation of renal cortical HS. Total renal cortical content of HS (A) and total sulfation of HS (B) were measured three times in pooled extracts from 5 rats per group (controls, high sodium rats 2 weeks and high sodium rats 4 weeks). Statistical differences between groups (N = 3/group) were tested using the Kruskall-Wallis test. *p<0.050.

Quantification of HS from renal cortex showed a small but significant increase after two weeks of high salt diet and a significant decrease upon four weeks high salt diet (both compared with 2 weeks high sodium and with baseline) (Fig 5A; all p = 0.05). Disaccharide profiling of renal cortical HS revealed a substantial increased sulfation after high salt feeding, both after 2 and 4 weeks (Fig 5B; both p = 0.05). From Table 2, where all individual disaccharides are depicted, it becomes clear that upon high salt feeding, all HS sulfation, including N-sulfation, 2-O-sulfation and 6-O-sulfation increased. This is mirrored by the mRNA expression values of the N- and O-sulfotransferases (Table 2) although differences among the groups did not reach statistical significance. From qRT-PCR analysis, data suggest a short up-regulation of 6-O-desulfating enzyme SULF2 at two weeks in the high sodium group, however, the structural analysis clearly indicate an increase of 6-O-sulfation upon high salt feeding, both at disaccharide and at 6-OST1 mRNA levels, especially at 4 weeks high sodium feeding (Table 2).

**Table 2:** Disaccharide composition of renal cortical HS chains and mRNA expression levels of enzymes involved in HS (de)sulfation.

Relative expression of disaccharides and of (de)sulfation enzymes	Control rats	Rats fed with high salt for 2 weeks	Rats fed with high salt for 4 weeks
**ΔUA-GlcNac**	41.6 [41.1–41.8]	36.8 [36.5–36.9]	38.2 [38.1–38.2]
**ΔUA-GlcNS**	18.7 [18.7–18.8]	20.2 [19.3–20.6]	18.9 [17.5–19.2]
**ΔUA-GlcNac.6S**	13.8 [13.8–14.1]	14.5 [13.9–14.7]	14.1 [14.1–14.2]
**ΔUA-GlcNS.6S**	6.1 [6.1–6.3]	7.1 [6.9–7.3]	6.6 [6.5–7.4]
**ΔUA.2S-GlcNac**	0.7 [0.7–0.8]	0.7 [0.0–0.8]	0.6 [0.4–0.8]
**ΔUA.2S-GlcNS**	10.9 [10.7–11.0]	12.7 [12.1–12.8]	13.2 [12.7–13.8]
**ΔUA.2S-GlcNS.6S**	8.2 [7.9–8.4]	8.7 [8.7–8.9]	8.6 [8.4–8.6]
**Total N-sulfation**	43.9 [43.7–44.2]	48.5 [47.9–48.8]	47.0 [47.0–47.3]
**Total O-sulfation**	48.0 [47.2–48.7]	51.9 [51.6–52.6]	51.4 [51.3–52.9]
**NDST1**	0.9 [0.5–1.9]	2.2 [0.6–22.2]	0.6 [0.2–71.6]
**2-OST**	0.8 [0.6–1.8]	1.6 [1.4–2.4]	1.1 [0.6–1.8]
**6-OST1**	0.9 [0.8–1.3]	1.0 [0.9–4.5]	1.2 [0.8–4.3]
**HSPE**	1.1 [0.3–1.5]	1.7 [1.6–5.8]	1.1 [0.8–3.2]
**SULF2**	1.1 [0.8–1.1]	1.3 [1.0–4.8]	1.0 [1.0–4.6]

Disaccharides are expressed as percentage of total disaccharides (N = 3/group of pooled kidneys). Enzymes involved in HS (de) sulfation (N = 5/group) are adjusted for GAPDH expression and expressed as fold increase compared to control rats (which mean value is set to one). Values are expressed in median [range]. Abbreviations used are frequently used in the proteoglycan field [19].

**Fig 6:**
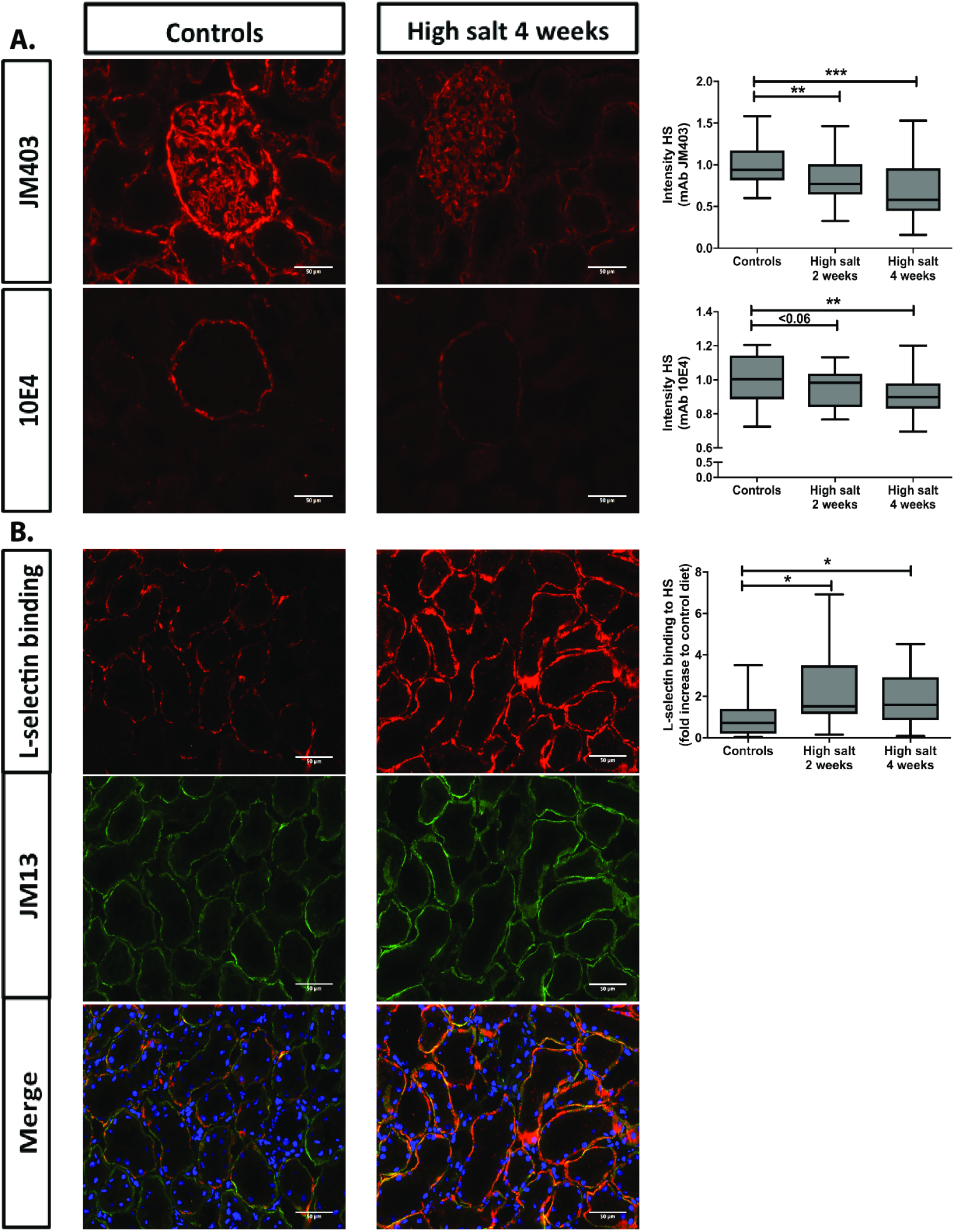
High dietary salt intake induced changes in staining intensity of anti-HS JM403 and 10E4, and HS-dependent L-selectin binding. Staining intensity is expressed compared to control group. L-selectin binding was done in a double staining with anti-HS mAb JM13, which is critically dependent on 2-O-sulfation. As can be seen, under high dietary salt conditions, staining intensity of L-selectin binding as well as JM13 increased, whereas anti-HS mAbs JM403 and 10E4 was partially lost, indicative for increased sulfation of HS under high dietary salt conditions. Scale bar represents 50 μm. Statistical differences between groups (N = 5/group) were tested using the Kruskall-Wallis test. *p<0.05; **p<0.01; ***p<0.0001.

**Fig 7:**
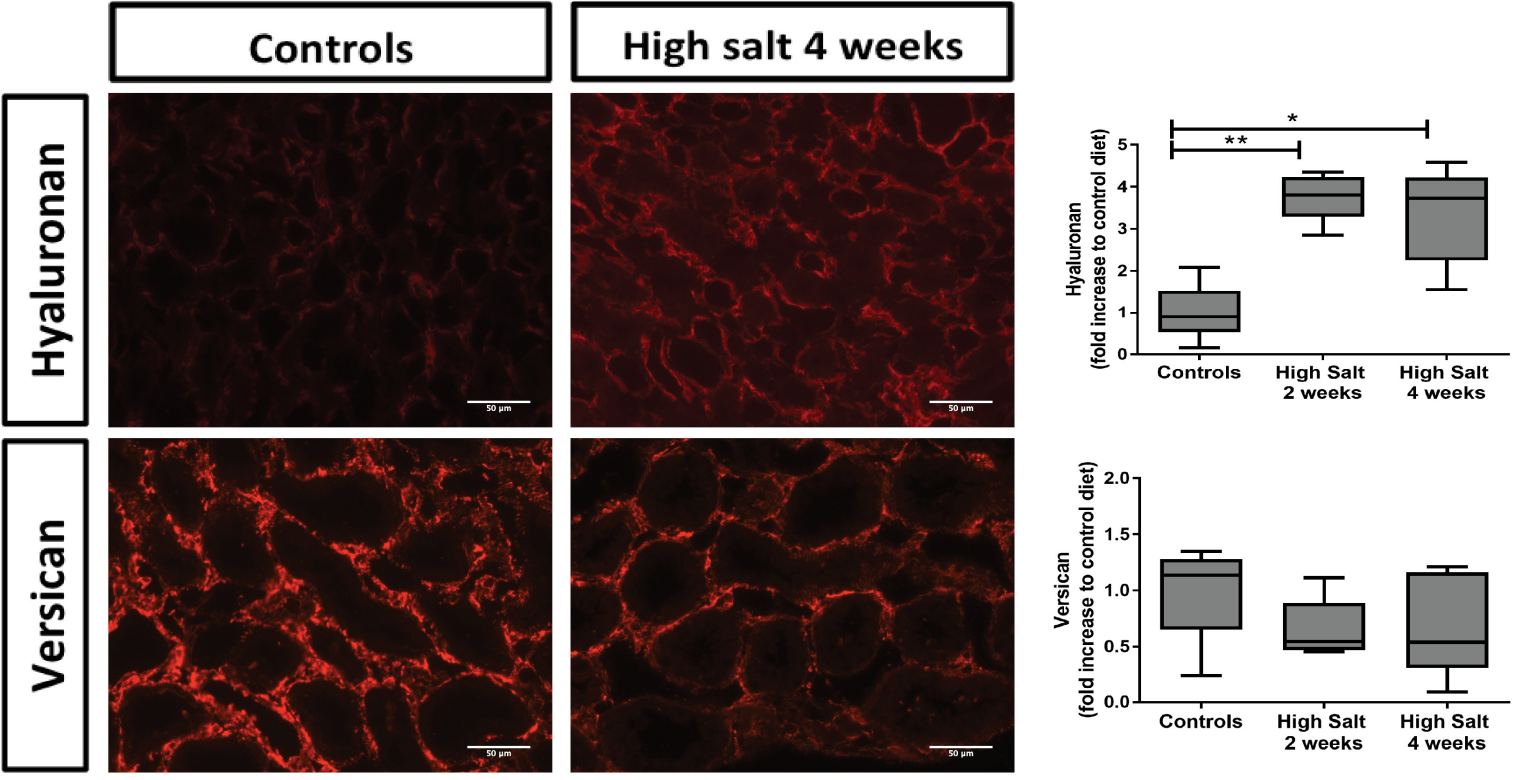
High dietary salt induced hyaluronan accumulation and apparent loss of versican. High dietary sodium increased hyaluronan especially in the cortico-medullary region (top), whereas versican tended to be reduced in the same areas (bottom). Scale bar represents 50 μm. Statistical differences between groups (N = 5/group) were tested using the Kruskall-Wallis test. *p<0.05; **p<0.01; ***p<0.0001.

Furthermore, we evaluated structural changes in renal HS by using monoclonal anti-HS antibodies and L-selectin and MCP-1 binding to HS followed by quantification (Fig 6). In previous studies, anti-HS mAbs JM403 and 10E4 are known to lose their intensity under different disease conditions. In high sodium fed rats, the glomerular staining intensity of JM403 was significantly decreased compared to controls at both two (p<0.01) and four weeks (p<0.001). For 10E4, the staining intensity of Bowman’s capsule decreased in high sodium rats as well compared to controls at both two weeks (p<0.06) and four weeks (p<0.01). Finally, we investigated the ability of HS to bind L-selectin and found that high sodium fed rats showed a significant increase of L-selectin binding compared to controls at both two (p<0.05) and four weeks (p<0.05). We found the same results for MCP-1 binding to HS (data not shown). Since the binding of L-selectin mainly depends on O-sulfation, we performed the L-selectin binding to heparan sulfate in a double staining with anti-heparan sulfate mAb JM-13, which is critically dependent on 2-O-sulfation [32]. Also JM-13 staining increased under high salt feeding conditions. Altogether, these data corroborate increased sulfation of renal cortical HS by high salt feeding. Next to heparan sulfate, we also evaluated and quantified tubulo-interstitial hyaluronan (by specific binding of biotinylated hyaluronan binding protein) and versican, an interstitial chondroitin sulfate proteoglycan. Interestingly, high salt diet also induced hyaluronan accumulation, maily in the cortico-medullary region (Fig 7; top; p<0.01). Versican, on the other hand seem to reduce by the high salt feeding conditions (Fig 7; bottom; NS). These data suggest, that high salt diet mainly induced increased heparan sulfate sulfation and hyaluronan content in the renal tubulo-interstitium.

### Associations between sodium excretion, renal tissue remodeling parameters and HS parameters

In order to investigate the chain of events and associations between changes in sodium excretion, changes in HS and tubulo-interstitial remodeling, we calculated Z-scores and correlated our findings (Table 3). In these analyses, we combined all three groups of rats. Urinary sodium excretion as a measure for salt intake correlated negatively with the intensity of 10E4 HS and positively with binding of L-selectin to HS. We next evaluated the association between HS changes and tubulo-interstitial remodeling. Loss of 10E4 and JM403 HS both correlated with an increased number of lymphvessels. Furthermore, JM403 intensity also inversely correlated with the influx of myofibroblasts and increased mRNA expression of collagen type III and MCP-1. The binding of L-selectin with HS correlated with lymph vessel density and mRNA expression of collagen type III and VCAM1. Finally, L-selectin binding was positively correlated with the influx of macrophages and increased expression of TGF-β. Finally, sodium excretion correlated with lymph vessel formation, and mRNA expression of collagen type III and VCAM1. Moreover, when adjusted for HS measures (10E4, JM403 and L-selectin binding), the association of sodium excretion with lymph vessels and VCAM mRNA expression was lost, however this was not the case for the association between sodium excretion and Coll III mRNA expression. This suggests that (part of) the correlation found between sodium excretion and both lymph vessel formation and VCAM1 mRNA expression is mediated via changes in HS function. However, the correlation between sodium excretion and collagen type III mRNA expression is independent of high sodium-induced HS changes.

**Table 3:** Correlations between sodium excretion (as a measure of sodium intake) and changes in HS (A), changes in HS and tubulo-interstitial remodeling (B), sodium excretion and tubulo-interstitial remodeling (C) and correlations of sodium excretion with tubulo-interstitial remodeling after partial correction for changes in HS (D).

**A. Sodium excretion correlates with changes in HS**		
*Variables*	***R***	***p-value***
Sodium excretion vs. L-selectin binding	0.586	0.022
Sodium excretion vs. 10E4	-0.674	0.006
**B. Changes in HS correlate with tubulo-interstitial remodeling**		
*Variables*	***R***	***p-value***
L-selectin binding vs. lymphvessels	0.549	0.034
L-selectin binding vs. macrophages	0.514	0.050
L-selectin binding vs. TGF-β mRNA expression	0.550	0.034
L-selectin binding vs. collagen type III mRNA expression	0.624	0.013
L-selectin binding vs. VCAM1 mRNA expression	0.689	0.004
10E4 vs. lymphvessels	-0.731	0.002
JM403 vs. lymphvessels	-0.547	0.035
JM403 vs. α-SMA	-0.607	0.016
JM403 vs. collagen type III mRNA expression	-0.774	0.001
JM403 vs. MCP-1 mRNA expression	-0.575	0.025
**C. Sodium excretion correlates with tubulo-interstitial remodeling**		
*Variables*	***R***	***p-value***
Sodium excretion vs. lymphvessels	0.774	0.001
Sodium excretion vs. collagen type III mRNA expression	0.654	0.008
Sodium excretion vs. VCAM1 mRNA expression	0.539	0.038
**D. Correlations of sodium excretion with tubulo-interstitial remodeling after partial correction for changes in HS (L-selectin binding, 10E4 and JM403)**		
*Variables*	***R***	***p-value***
Sodium excretion vs. lymphvessels	0.520	0.083
Sodium excretion vs. collagen type III mRNA expression	0.669	0.017
Sodium excretion vs. VCAM1 mRNA expression	0.316	0.317

Spearman Rank correlation was performed on the Z-score of the variables in all rats (N = 15) and p<0.05 is considered to be a significant correlation.

## Discussion

For the first time, we have shown that a high sodium diet increases sulfation of renal HS in salt-insensitive, normotensive healthy rats, and is associated with renal tissue remodelling events. Interestingly, these tubulo-interstitial changes are not associated with increased sodium content of the kidneys.

After receiving a high sodium diet for 1 week, the rats showed a marked increase in blood pressure. However, this peak was transient and the blood pressure dropped to control values after two weeks. Evaluation of the blood pressure over time by determining and comparing the AUC per group, revealed that the rats in our experiment were capable of maintaining a stable blood pressure during the course of the study, independent of the sodium content of their diet. Our data showed that they compensated this sudden increase of sodium intake by increasing their sodium excretion, water intake and urine production. In addition to the stable blood pressure, plasma sodium concentrations remained constant in both controls and high sodium rats. Studies by the group of Titze suggested that sodium could be stored non-osmotically as glycosaminoglycans in the skin. In this way, the skin can function as an extra buffering compartment for sodium in the body, in order to compensate for high sodium intake [13,15,33]. However, this non-osmotic sodium storage had not been investigated in the kidney yet. We therefore measured renal tissue sodium concentrations and compared these to dermal tissue sodium concentrations as a positive control. We measured dermal sodium content in both ear skin and abdominal skin, since studies have shown differences in distribution of sodium throughout the body [34]. In both ear and abdominal skin we confirmed a significant increase in the sodium concentration over time. However, we did not find this for renal sodium concentrations. This indicates that non-osmotic sodium storage does not occur in the kidney. A possible explanation might be that HS is the predominant GAG present in kidney tissue, while hyaluronan, chondroitin sulfate and dermatan sulfate are the important GAGs in the skin [35], suggesting that the process of non-osmotic sodium storage may be specific for certain GAGs present in various tissues.

Interestingly, despite the fact that HS did not show increased sodium binding properties, the high salt diet has profound effects on the amount of HS expressed in the kidney and the degree of sulfation. When we analyzed the polysaccharide sulfation patterns after the different diets, we found that *N*-sulfation, 2-*O*-sulfation and 6-*O*-sulfation increased in high sodium rats. These findings were also reflected by the expression values of the *N*- and *O*-sulfotransferases. Previous studies clearly showed close ties between 6-*O*-sulfation, 6-O-sulfotransferases and the sulfatases Sulf1 and Sulf2 [36–40]. In this study, we found an up-regulation of 6-*O*-desulfating enzyme Sulf2 at two weeks; however, the structural analysis clearly indicated an increase of 6-*O*-sulfation upon high salt feeding after 4 weeks, which could suggest a delayed effect or a restoration of function. Lamanna et al. showed through knockout models that loss of Sulf2 caused a decrease in N- and 2-*O*-sulfation, while loss of Sulf1 had no significant effect. Furthermore, loss of both Sulfs resulted in an increase in these sulfate moieties [41]. This suggests a dynamic interplay between Sulf activity and the HS biosynthetic machinery, which is not completely understood yet.

While changes in the disaccharide composition are apparently modest in our high sodium rats compared to their controls, the functional consequences of seemingly small structural changes and an increased sulfation of HS can be critical [19]. Next to the analysis of the disaccharide composition, anti-HS antibodies can be used as an alternative way to study the fine structure of HS. Previous studies by our group, showed that a decreased staining intensity with anti-HS antibodies, specifically for the JM403 and 10E4 epitopes, are associated with increased renal damage and worsening of disease conditions [21,42,43]. In this study we used three different antibodies/L-selectin to profile changes in heparan sulfates. We used anti-heparan sulfate mAb JM403 of which its specific epitope is dominated by an N-unsaturated GlcN unit (free amino group) in low sulfated heparan sulfates [42,44]). JM-403 strongly recognizes heparan sulfates in the glomerular basement membranes, in Bowman’s capsule, and in some tubular en endothelial basement membranes. The epitope of mAb 10E4 is characterized by a mixed N-acetylated/N-sulfated sequence in low sulfated heparan sulfates, mainly present in Bowman’s capsule and some tubular basement membranes [42]. Both mAbs JM403 and 10E4 loose reactivity with heparan sulfates upon increased sulfation [42]. Finally, we demonstrate heparan sulfate by its interaction with recombinant L-selectin-IgM. The binding of L-selectin with heparan sulfate is critically dependent on (6-)O-sulfation [45]. Based on the characterization one can predict that upon increased sulfation of heparan sulfates, the binding of JM403 and 10E4 will decrease, while the binding of L-selectin with heparan sulfates will increase as what happened in our study (Fig 6). In order to investigate the binding of L-selectin to sulfated HS we also performed a double staining of anti-heparan sulfate mAb JM13 (which is critically dependent on 2-O sulfation as shown by *van den Born et al*. [42] with the L-selectin binding assay. It shows co-localization in tubular basement membranes as has been shown before by our group [26,45]. Consequently, we show that the increased binding of L-selectin to heparan sulfates after high salt diet is accompanied by an increased JM13 staining intensity, which is an extra prove of increased sulfation of heparan sulfate upon high salt diet.

Previous studies in more severe models for kidney damage, showed that changes in L-selectin and MCP-1 binding can lead to influx of immune cells and tubulointerstitial remodeling, however HS changes secondary to high sodium intake have never been studied before. [22,46–48]. In order to investigate the effects on proteoglycans other than HS, we investigated hyaluronan and versican. Hyaluronan is frequently used as a marker for water handling by the skin because of their significant water-binding capacity and we show that a high sodium diet, also leads to higher expression of hyaluronan [49]. On the other hand, versican shows no significant change over time while it has been known to be associated with lymphangiogenesis and tissue remodeling [49,50] during regeneration and renal transplantation. Apparently, the GAG/proteoglycan response to various noxi is not uniform, rather context dependent.

HS is found in the matrix (mainly basement membranes) and on cell surfaces [19]. In our analytical renal HS analysis (disaccharide composition) we purified HS from kidney homogenates, so did not discriminate between matrix and cellular HS. Multiple studies however, have shown that the majority of HS is found in basement membranes [42,44,51]. This is also corroborated by our HS visualization in Fig 6 where anti-HS mAb JM403 stained the glomerular basement membranes (and some other basement membranes), anti-HS mAb 10E4 stained Bowman’s capsule (and some other basement membranes). The double staining of anti-HS mAb JM13 with L-selectin binding to HS, shows that JM13 and L-selectin both recognize HS mainly in tubular basement membranes. These data do not deny the presence of extracellular surface layer (ESL) HS, rather demonstrate that by far the majority of renal tissue HS is in the basement membranes and that the cell surface HS is below detection limit of the immunohistochemistry procedures. We previously showed cellular HS on tubular epithelial cells in renal tissue of transplant recipients using anti-HS mAb 10E4 [52], and on the apical cell membranes of hepatocytes in liver tissue, by various anti-HS mAbs [43], which shows that cellular HS can be demonstrated by anti-HS mAbs. So, based on these considerations, we conclude that our data (analytical HS analyses and immunofluorescence stainings with anti-HS mAbs and L-selectin) refers largely to matrix-associated (= basement membrane-associated) HS. In our study, no albuminuria is evoked, suggesting that the glomerular filtration barrier is not adversely influenced by high salt intake.

These changes in HS function and structure were already found after 2 weeks, while changes in tubulo-interstitial remodeling (i.e. influx of macrophages, T-cells and myofibroblasts and increased lymph vessel formation) became apparent only after 4 weeks. On an mRNA level, VCAM1, MCP-1, TGF-β and collagen type III expression increased after 2 weeks, VCAM1 and TGF-β normalized after 4 weeks. Also the mRNA expression levels of most HS sulfotransferases peaked at 2 weeks high sodium diet, and reversed thereafter. Therefore, the changes in renal HS (mainly at week 2) and the tubulo-interstitial remodeling responses on a mRNA-level (also at week 2) could be induced by the peak in blood pressure at week 1, and in their turn led to tubulo-interstitial remodeling on a tissue level after 4 weeks. We therefore speculate that the high sodium diet-induced changes in renal HS structure and tubulo-interstitial remodeling could be transient in normotensive rats, as we showed that the rats could compensate for a rise in blood pressure by increasing their sodium excretion and their non-osmotic dermal sodium storage. Importantly however, this also suggests that if this process is disturbed in salt-sensitive rats and patients, high dietary salt intake could lead to progressive renal damage from 2 to 4 weeks onwards. An alternative blood pressure-independent explanation for the renal damage found after the high sodium intake in our study can possibly be found in the studies of *Oberleithner et al*., where they show that only a small increase of the plasma sodium concentration (5%) is enough to stiffen the endothelial cells by 25%, leading to cellular dysfunction. Next to this, it has been demonstrated by surface measurements that the endothelial glycocalyx detoriates when sodium is elevated [53–55]. The concentration in our study of 8% sodium in the diet is very high, however did not lead to observable adverse reactions to the diet by the rats. Moreover, the high sodium diet did not lead to a lower body weight. It might be that exposure to lower sodium content in the diet finally lead to the same finding, eventually after longer time frames.

Finally, it was interesting to investigate potential links between all phenomena described. We see a clear time-dependent association between sodium excretion as a reflection of sodium intake and workload for the kidney, functional changes in HS and tubulo-interstitial remodeling events, (lymphangiogenesis and mRNA expression of collagen type III and VCAM1), which is being covered by statistical correlation. To correct for confounders, partial correlation was performed, which confirmed that at least VCAM1 mRNA expression and lymphangiogenesis increases when the sodium excretion increases, which is probably due to changes in HS function, since its significance disappeared after correction. Previous studies by our group in different disease models have shown that conversion of heparan sulfate into pro-inflammatory glycans can conttribute to renal tissue worsening. Upon renal ischemia/reperfusion, subendothelial heparan sulfates become converted into glycans able to bind L-selectin and MCP-1 and contribute to the influx of macrophages into the kidney [46]. In another study we showed that upon renal transplantation, vascular and glomerular heparan sulfates convert into profibrotic glycans that bind FGF2 and contribute to glomerulosclerosis and neointima formation [56]. In a third study, we showed that in mice which were knocked down for the basement membrane associated heparan sulfate proteoglycans Coll XVIII and Coll XV (which are hybrid collagen/proteoglycan molecules) the influx of neutrophils and macrophages in response to renal ischemia/reperfusion was completely prevented, and renal function (serum urea) and renal tubular injury was significantly improved [57]. In the last study we also provided extensive in vitro evidence that the MCP-1 driven migration of monocytes is crucially dependent on heparan sulfates. Studies have previously shown links between changes in HS function and increased proliferation and influx of fibroblasts and lymphocytes, however, for the first time we show that an increased sodium excretion could induce pro-inflammatory conversion of HS, ultimately contributing to tubulo-interstitial remodeling [21,58,59].

At present, mechanisms by which high dietary sodium increased renal HS sulfation remain unknown. To evaluate a direct sodium effect on renal HS and tissue remodeling, we conducted an *in vitro* study on precision-cut mouse kidney slices and titrated different sodium concentrations into the medium [60]. We did not see any changes in VCAM1, MCP-1, SULF2 and TGF-β mRNA expression levels at 24 hours after the addition of 40 and 80 mmol NaCl compared to controls (two independent experiments in triplicate; data not shown). This suggests that the sodium effect on these markers as observed *in vivo* (Fig 3 and Table 2) thus might be influenced by physiological processes. We speculate that high working load of proximal tubular epithelial cells, increased RAAS and/or sympathetic activity, increased release of vasoactive mediators such as endothelin and vasopressin, or cytokine release by inflammatory cells might play a role, however this has not been studied in detail yet.

In conclusion, we deliver here a detailed description of a mechanistic pathway, in which a high sodium intake by healthy normotensive rats converts renal HS into high sulfated pro-inflammatory glycans. HS is not involved in sodium binding, however the modifications to HS caused by the high sodium diet are associated with tubulo-interstitial remodeling in the kidney. We also provide further evidence to the emerging view that blood pressure independent mechanisms also play an important role in aggravation of renal diseases by high dietary sodium intake. Further research will be needed to investigate the exact mechanism leading to the conversion of HS into pro-inflammatory glycans by a high sodium diet. Since these data show harmful renal effects of excess dietary sodium, most likely independent of blood pressure, we propose better monitoring of sodium intake and sodium excretion in renal patients independent of their response to blood pressure medication. Furthermore, our study underlines the importance of limiting sodium intake in healthy persons as well, since the effects of high sodium intake could already been seen after a 4 week high salt diet in otherwise healthy rats. This could be a highly relevant decree for the prevention of renal disease in general population.
